# Inversion of Soil Organic Matter Content Based on Improved Convolutional Neural Network

**DOI:** 10.3390/s22207777

**Published:** 2022-10-13

**Authors:** Li Ma, Lei Zhao, Liying Cao, Dongming Li, Guifen Chen, Ye Han

**Affiliations:** 1 College of Information and Technology, Jilin Agricultural University, Changchun 130118, China; 2 Institute for the Smart Agriculture, Jilin Agricultural University, Changchun 130117, China; 3 Institute of Technology, Changchun Humanities and Sciences College, Changchun 130118, China

**Keywords:** deep neural networks, soil organic matter, intelligent agriculture, remote sensing

## Abstract

Soil organic matter (SOM) is an important source of nutrients required during crop growth and is an important component of cultivated soil. In this paper, we studied the possibility of using deep learning methods to establish a multi-feature model to predict SOM content. Moreover, using Nong’an County of Changchun City as the study area, Sentinel-2A remote sensing images were taken as the data source to construct the dataset by using field sampling and image processing. The LeNet-5 convolutional neural network model was chosen as the deep learning model, which was improved based on the basic model. The evaluation metrics were selected as the root mean square error (RMSE) and the coefficient of determination R^2^. Through comparison, the R^2^ of the improved model was found to be higher than that of the linear regression method, Support Vector Machines (SVM) (RMSE = 2.471, R^2^ = 0.4035), and Random Forest (RF) (RMSE = 2.577, R^2^ = 0.4913). The result shows that: (1) It is feasible to use the multispectral data extracted from remote sensing images for soil organic matter content inversion based on the deep learning model with a minimum RMSE of 2.979 and with the R^2^ reaching 0.89. (2) The choice of features has an impact on the prediction of the model to a certain extent. After ranking the importance of features, selecting the appropriate number of features for inversion provides better results than full feature inversion, and the computational speed is improved.

## 1. Introduction

Soils play a key role in agriculture. Therefore, the management of cultivated soils can guarantee the sustainable productivity of cultivated land. In the planning of cultivated land, the selection of crops should be based on the characteristics of the soil. On the other hand, appropriate crop selection can also modify the soil and improve the ecological environment [1,2]. Carbon is stored in the soil as organic matter, and the main sources of organic matter come from the natural degradation of plants or animals, and the decomposition and synthesis of microorganisms [3]. Soil organic matter (SOM) is an important component of soil and is the main source of nutrients required for crop growth [4,5]. Moreover, it is related to many properties of the soil, such as structure, aeration, fertility, and buffering properties [6]. Besides the effect of other factors, the organic matter content has a direct influence on the level of soil fertility [7]. Therefore, it is important to understand the SOM content of different lands for the modification or utilization of cultivated lands. It can also improve the utilization rate of cultivated land resources and guide the management of agriculture [8].

The traditional method used to determine SOM content usually requires soil samples to be collected in the field and sent to the laboratory for testing after being processed [9]. Though this method has a high accuracy of measurement, it has the disadvantages of heavy manual labor, a long data collection period, and low measurement efficiency. Previous experiments have shown that the organic matter in the soil can affect the spectral performance of the soil [10]. Compared with the traditional method, the inversion of SOM content by using spectral data is characterized by the advantages of faster speed, lower energy consumption, and broader coverage [11,12]. Gu et al. [13] studied the Beijing municipality based on wavelet transform technology and found that high-frequency information in the near-infrared region (NIR) is more sensitive to SOM content; Zhai [14] used GF-1 and Landsat-8 as data sources to study the wetland soil in Anyi County and the Gao’an Research Area based on the multiple regression method and found that the Band 5 (850 nm) reflectivity of Landsat-8 images has the strongest correlation with SOM content; Liu et al. [15] used Sentinel-2A and Landsat 8 images as data sources and filtered the spectral bands by using a random forest algorithm to establish the SOM inversion model. Through the study of the soil of Shengli Farm in Heilongjiang Province, the response bands of the SOM spectra were found to be Band 3 (560 nm), Band 4 (660 nm), Band 6 (740 nm), and Band 8 (850 nm), respectively.

The common spectral images used for SOM content inversion are hyperspectral images and multispectral images. The former contains hundreds of narrow bands with much wider spectral details than multispectral images [16,17]. However, the publicly and freely available hyperspectral images are usually and only available for a specific small area, which cannot be flexibly selected based on the study area and soil sampling time of the experiment. In addition, the spectral data must also be reduced because of a variety of factors, including the environment and instruments [18].

Multispectral images are typically taken by remote sensing, and, when compared to hyperspectral images, the multispectral image of the sampling region can be acquired more quickly and flexibly [19]. Currently, in most studies of the multispectral data SOM content inversion, Landsat images are used [20]. In this case, in this experiment, we try to use Sentinel-2A satellite images as the data source. Sentinel-2 satellite was launched in 2015, and can display 13 spectral bands with a wide swath width of 290 km. The resolutions of the images vary with the bands, including the ground resolutions of 10 m, 20 m, and 60 m [21,22]. Lu et al. [23] found that the SOM-sensitive band ranges from 650–750 nm, and Sentinel-2A images contain data in three bands within this range; in contrast, Landsat images include one band only. However, the spectral features of soil water content and iron oxide content overlapped with those of the SOM content, resulting in a higher difficulty in the inversion of SOM content. The environmental factors at the collection time also have an influence on the spectral performance of the SOM content [24]. It is known from previous studies that the response of the spectral index to SOM can be effectively improved by the mathematical transformation of the spectral index obtained, and this improvement is visualized by the numerical importance of the features [13,25].

There have been many previous modeling experiments conducted using deep learning in the study of SOM content inversion from hyperspectral images [26,27]. However, the inversion of SOM content from a multispectral image has mostly been conducted by using statistical methods to model the individual features obtained from a single band or through a combination of multiple bands due to the small number of bands [28]. In this paper, we try to use the deep learning method to model the obtained spectral data, thereby achieving the inversion of SOM content. We aim to make the feature dimension of the model more complete by increasing the number of features. Deep learning is available for the automatic completion of feature extraction by computer, which has a higher accuracy for solving complex problems [29]. At present, deep learning methods are applied in automated pest detection [30], crop disease detection [31], and crop classification [32]. The Convolutional Neural Network (CNN) is a type of deep learning model, and, compared with other deep learning models (such as multi-layer perceptron, recurrent neural network, etc.), CNN is good at extracting data features with strong learning ability. At the same time, it has the advantages of high fault tolerance, good adaptivity, and fast operation speed [33,34]. Due to the small number of samples, training with a neural network that is too deep is prone to overfitting; therefore, a shallow layer network is selected in this paper. The LeNet-5 network is chosen as the base model for this experiment due to its good performance in regression, clear structure, small size, and fast operation speed [35].

In this study, multispectral data extracted based on Sentinel-2A satellite images and SOM content data detected by field sampling are used as data sources. The spectral data are processed by mathematical transformation and a neural network approach is used for the feature selection of the whole data. We construct a soil organic matter content inversion model based on a 1D CNN and implement the feature extraction of a large number of features by computer. The root mean square error (RMSE) and the coefficient of determination R^2^ are used as evaluation indicators to quantify the prediction effect of the model. This study aims to use free multispectral data to model SOM content through a deep learning approach to obtain SOM content under low-cost conditions.

## 2. Materials and Methods

### 2.1. Materials

#### 2.1.1. Study Area

In this experiment, Nong’an County was taken as the study area, which is near Changchun City and is located in the hinterland of Songliao Plain. The highest point in the area, Ximajia, is located in the northwest, with an elevation of 219 m. The lowest point, the Yitong River Exit, is located in the northeast, with an elevation of 160 m. The geographic location is 124°31′–125°45′ E, 43°55′–44°55′ N. Nong’an County has an average annual temperature of 4.7 degrees, a frost-free period of 145 days, 507.7 mm of precipitation, and an effective cumulative temperature of 2800 degrees [36].

The research region has a wide range of soil types, and the topographic soils include black soils (relatively moist soils with high organic matter content) and black calcium soils (distributed in semi-arid areas with low organic matter content). Local soils include meadow soils, alluvial soils, saline soils, marsh soils, and wind–sand soils [37].

#### 2.1.2. Acquisition and Processing of Soil Samples

The collection of soil samples was completed in 2017, and the coordinate locations of the sampling sites were recorded during sample collection in order that, in the subsequent experiments, it is possible to locate them on remote sensing satellite images. A total of 800 soil samples were collected based on a five-point sampling method. Specifically, five samples of soil mixed in the four corners around the sampling site and the center were taken, and part of the soil was collected into a sealed bag for storage. The soil samples were then spread out in a ventilated place and air-dried for one week. After drying, the soil samples were ground in a ceramic mortar and filtered through a 60-mesh sieve. The SOM content of the samples was determined by using the potassium dichromate–sulfuric acid method [9]. After determination, the organic matter content of the sample soils ranged from a minimum of 14.6 g/kg to a maximum of 36.65 g/kg, and most of the soil samples were tertiary soils (organic matter content of 20–30 g/kg).

**Figure 1:**
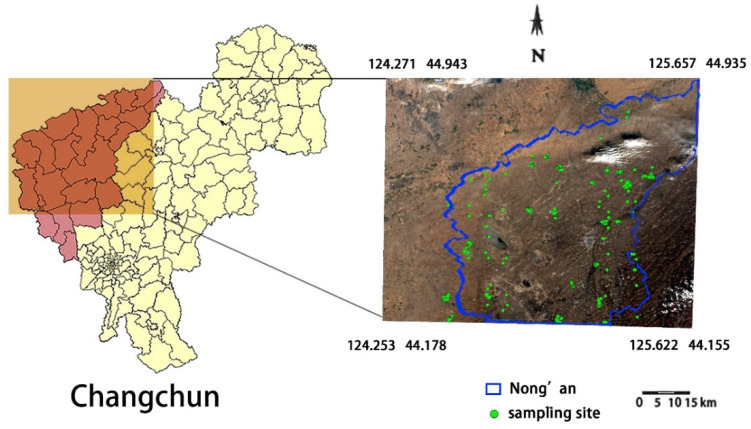
Distribution of soil sampling sites in the study area (the blue area refers to the administrative district of Nong’an County).

The soil samples collected include light loess saline black calcium soil, medium rotten loess black soil, thin rotten loess meadow black calcium soil, medium rotten loess calcareous black calcium soil, thin rotten loess calcareous black calcium soil, thick rotten loess calcareous black calcium soil, loamy calcareous alluvial soil, thin rotten loess calcareous black calcium soil with a red clay bottom, thick rotten fixed meadow sandy soil, and thin rotten loess calcareous black calcium soil with a red clay bottom. The distribution of soil sampling points is illustrated in Figure 1.

#### 2.1.3. Remote Sensing Image Processing

Since the soil samples were collected in 2017 during the bare soil period, the remote sensing image captured using Sentinel-2A on 29 April 2017 (bare soil period) was selected for the study, which was basically the same as that at the time of soil collection and without vegetation cover. Moreover, the conditions in the sampling area were favorable, the picture cloudiness was low, and the remote sensing image could reflect the surface data at the time of shooting.

Sentinel-2A images were downloaded from the ESA Copernicus Data Centre (https://sentinel.esa.int/web/sentinel/home, accessed on 6 November 2021), and the spectral characteristics of the Sentinel-2 images are shown in Table 1. The images downloaded from the website are orthorectified and geometrically refined only; therefore, it is necessary to conduct additional processing of the images by using the processing software SNAP [38].

**Figure 2:**
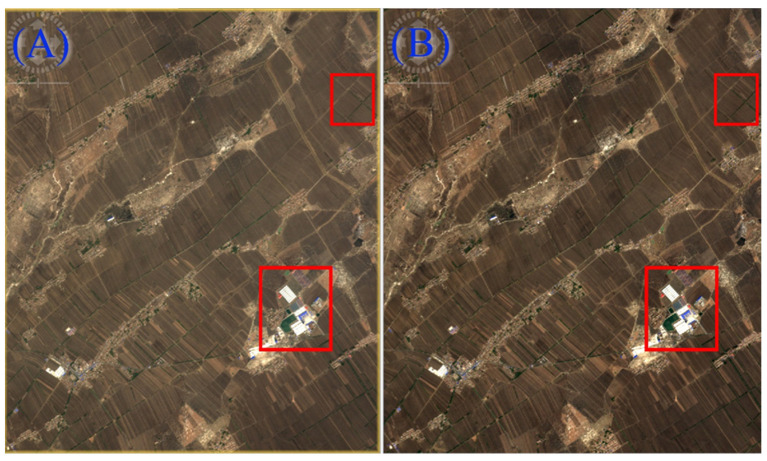
Sentinel image comparison before and after processing: (**A**) before image processing; (**B**) after image processing. The contrast of image resolution changes in the red box is obvious.

The first step includes the atmospheric correction of the image to mitigate the influence of the atmosphere on the image, the inversion of the true surface reflectance of the feature, and the conversion of the radiant brightness value to the actual surface reflectance by using the plug-in Sen2Cor. To further improve the spatial resolution of remote sensing images, the original 60 m- and 20 m-band images were synthesized into 10 m-band images with the Sen2Res plug-in. Figure 2 compares sentinel photos from a specific sampling area before and after processing. It can be seen that the boundary between lands is clearer and the spatial resolution is improved after processing. Since SNAP software cannot be used to directly write multi-size data and convert the format into ENVI, the processed images are resampled by using the resampling function, and then output in ENVI format. To facilitate the subsequent extraction of surface reflectance, the exported images of each band are synthesized into a single multi-band image using the Layer Stacking tool of the software ENVI. Based on the coordinates recorded during field sampling, the image was opened in ENVI Classic, and the surface reflectance of each band at the sampling point was extracted [39]. Since the coordinates of each image element in the image are calculated according to the coordinates of the center point, the extracted coordinate data may not match with the input measured coordinate data, and the principle of proximity is adopted to match the closest point to the sampled point as the output data. Some of the measured points are too close, and there may be only one derived value. Therefore, the points far away from the center point are discarded. Moreover, the sampling points covered by clouds and located around the clouds, which were affected by the environment, were deleted from the image. In total, there were 563 sampling points retained after selection by analyzing the remote sensing images. The obtained spectral data and the measured SOM content data were processed by coordinates, and the dataset was constructed and divided into the training set and test set. The processed data can be found in Table S1.

The association between surface reflectance and SOM content can be strengthened based on mathematical transformation, and the useful information can be enhanced [14]. In this paper, mathematical transformations, such as difference value (Ri − Rj), ratio (Ri/Rj), inverse (1/R), and logarithm (lnR), were applied to the extracted surface reflectance to augment the data. The normalized difference vegetation index (NDVI) is primarily used to judge the vegetation growth status based on the intensity of the NIR reflected. It was found in the previous studies that the response of SOM spectra in NIR was also very sensitive. Therefore, we tried to use NDVI as one of the mathematical transformation methods. The relevant formula is shown in Equation (1), where NIR refers to the near-infrared band and R represents the red band.

(1) $$\mathrm{NDVI}=\frac{\left(\mathrm{NIR}\;-\;R\right)}{\left(\mathrm{NIR}\;+\;R\right)},$$

Since band 10 reflected the atmospheric band, it was automatically removed during the atmospheric correction. Band 1 was aerosol; therefore, it was also removed. After data processing, we obtained 11 bands of surface reflectance R, 11 log-transformed features, 11 inverse-transformed features, 50 difference-transformed features, 50 ratio-transformed features, and 19 NDVI-processed features, totaling 152 features.

### 2.2. Methods

#### 2.2.1. Inversion Model of Soil Organic Matter Content

**Figure 3:**
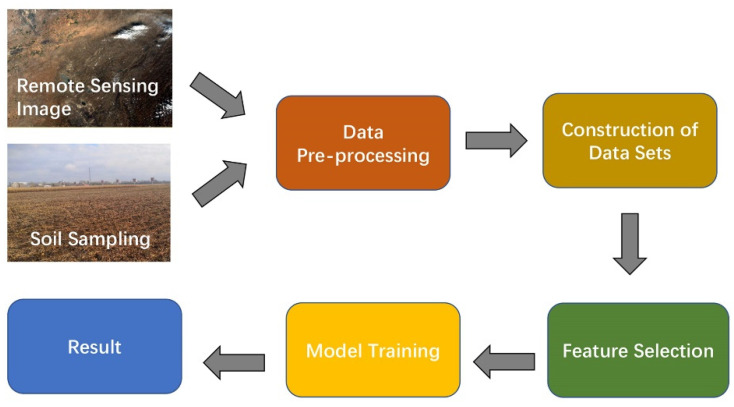
Flow chart of the experimental model.

The workflow of the inversion experiment on SOM content is shown in Figure 3. After acquiring remote sensing image data as well as the field soil sampling and assay data, respectively, the data need to be pre-processed by using SNAP and ENVI software [39,40], thereby improving the quality of remote sensing images and mitigating the influence of the environment on the images. The spectral information of the sampling points contained in the remotely sensed image is extracted based on the geographic coordinates and is matched with the SOM content data of the sampling points obtained from the field sampling and assay [41]. The features are expanded by mathematical transformation to construct the Spectral-SOM dataset. Some features with low importance are filtered out via feature selection in order to prevent the dataset from becoming noisy due to the overly rapid expansion of the number of features. The data were input to the backbone network for training, and the prediction results of the model were obtained.

#### 2.2.2. Feature Selection

After the mathematical transformation, features with low sensitivity to SOM are also generated in the data, amplifying the noise of the data. In feature selection, the characteristics are ranked according to importance, and the features with a higher value are chosen. Through feature selection, the data can be downsized to reduce overfitting situations, thereby minimizing the model input and strengthening the generalization ability of the model. Some scholars have experimentally demonstrated that optimizing the input quantity is beneficial to improve the stability and accuracy of the SOM content inversion model to some extent [15]. In this experiment, feature selection relies on the multi-layer perceptron (MLP) algorithm to retain the features sensitive to SOM content and to reduce invalid features.

In this experiment, the importance of features is ranked through the importance analysis of independent variables in the MLP algorithm [42,43]. This was achieved by using the multi-layer perceptron algorithm in the SPSS analysis software neural network and by setting the SOM content as the dependent variable and feature values as the independent variables. The dataset was divided into a training set and a validation set at a ratio of 7:3. The hidden layer of MLP was set as two layers, and there were two units in each layer. The activation function is the tanh function. After the running of the algorithm, the importance analysis of the algorithm for the input features is implemented according to the weights assigned to different features.

#### 2.2.3. Backbone Network

**Figure 4:**
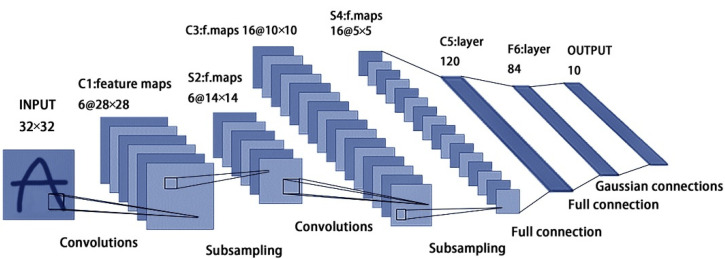
LeNet-5 network model structure.

The LenNet-5 is a classical convolutional neural network proposed by Yann Lecun [44], which was originally developed for handwritten character recognition with obvious results. Later, it was improved and applied to many fields. Without considering the input layer, the classical LenNet-5 CNN is divided into seven layers, and the network structure is shown in Figure 4. The layers of C1 and C3 are convolutional ones responsible for feature extraction, while the layers of S2 and S4 are pooling ones, also known as downsampling layers, which are used to reduce the number of parameters and computation. The C5 layer is essentially a convolutional layer. However, its convolutional kernel size is 5 × 5, and the feature map size of the fourth pooling layer is also 5 × 5; therefore, the feature map size of the C5 layer becomes 1 × 1, constituting the full connection between the S4 layer and the C5 layer. The F6 layer is fully connected to the C5 layer, which outputs the feature map to the output layer. The last output layer outputs a tensor with a length of 10.

#### 2.2.4. Improved Model

**Figure 5:**
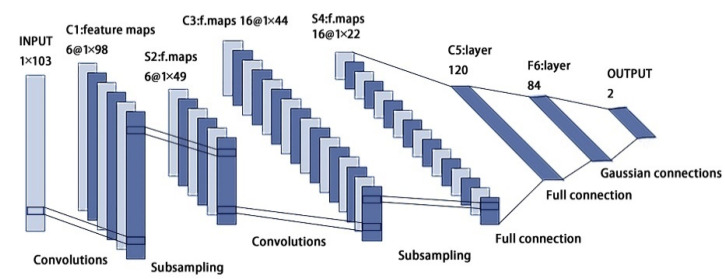
1D LeNet-5 network model structure.

The experimental model was modified based on the LeNet-5 convolutional network model by using deep learning ideas. Since the input data are the surface reflectance of different bands and those after mathematical changes, and because both of them are one-dimensional data, we used the one-dimensional convolution kernel for convolution operation. The model structure is shown in Figure 5. In the establishment of the model, a 1D convolutional layer and a 1D pooling layer were chosen.

**Figure 6:**
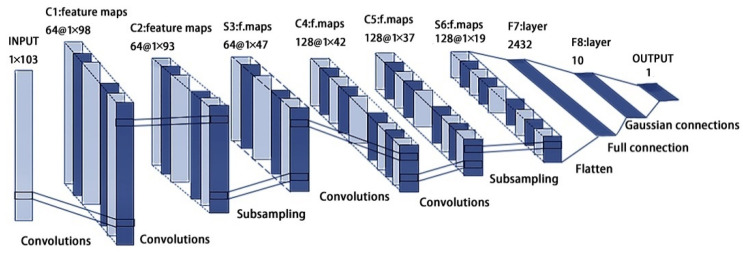
Improved LeNet-7 network model structure.

In order to make the model more adaptable to the dataset, further improvements were made based on the 1D model, and the model structure is shown in Figure 6. The specific improvement points were: (1) Due to a large number of features in the dataset, the original network has too much data in the input to the full connection layer, therefore the depth of the model needs to be increased. However, the deeper the network, the greater the computational effort, and the more likely it is to produce overfitting. After experiments, two convolutional layers were added to the original model. (2) Since the size of the data output from the S6 layer is 128 × 19, and the dense layer cannot perform 2D operations, the C7 layer was changed to the flattened layer. The flattened layer can make the multi-dimensional input 1D, thus realizing the transition from the convolutional layer to the full connection layer. (3) To improve the learning ability of the model, the number of convolutional kernels is increased. The number of convolutional kernels is increase from 6 to 64 for layers C1, C2, and S3, and from 16 to 128 for layers C4, C5, and S6. In addition, since the number of samples is not large, a 30% dropout is added after the flattened layer to prevent overfitting.

## 3. Experimentation

### 3.1. Computational Hardware and Platform

The experiments in this paper were conducted on a Lenovo Legion R7000P2021H computer. The computer consists of an AMD Ryzen 7 5800H with Radeon Graphics CPU with a clock speed of 3.20 GHz, 16.0 GB RAM, 512 GB hard disk, and a NVIDIA GeForce RTX 3060 Laptop GPU. The operating environment is Windows 10, TensorFlow = 2.1.0, Python 3.6.

### 3.2. Feature Selection Based on Feature Importance

**Figure 7:**
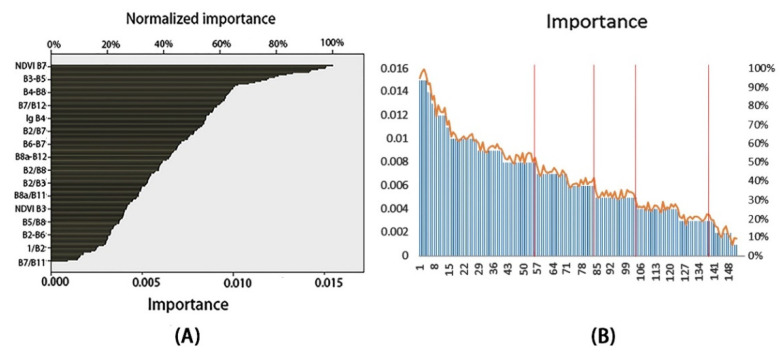
Feature importance ranking: (**A**) importance analysis of independent variables for multi-layer perceptron (MLP) algorithm; (**B**) number of features in each importance class (the orange line is the normalized importance curve; the red line is the point where the importance of the feature decreases significantly).

Using the originally constructed dataset as the input, the importance of each feature is ranked by using the MLP algorithm; moreover, it retains the more important features. The results of the algorithm operations are shown in Figure 7. Due to the large number of features involved in the ranking, unlike other studies that ranked features in groups for each mathematical transformation obtained, the ranking was performed as a whole. In general, the results obtained from the importance ranking are consistent with those from previous studies (due to the different division of wavelengths between Landsat-8 and Sentinel-2A images, the correlation between the bands of the two types of images should be found based on the wavelengths. For example, Band 5 of Landsat-8 corresponds to Band 8 of Sentinel-2A) [13,15,25].

It can be seen from Figure 7B that the features exhibit a significantly decreasing trend at some nodes, such as 29, 57, 84, etc. To describe the influence of the number of features on the inversion model of SOM content, this experiment was designed to use different numbers of features for inversion. To satisfy the requirement of the deep model, five types of experiments based on 55 features, 84 features, 103 features, 139 features, and 152 features, respectively, were set up according to the decreasing trend of feature importance, as shown in Figure 7.

### 3.3. Model Training

The improved model of both the C1 layer and C2 layer consists of 64 channels, with a convolutional kernel size of 1 × 5, which is utilized for feature extraction. ReLU is used as the activation function, characterized by very fast computation and convergence. The S3 layer consists of 64 channels with the pooling kernel size of 1 × 2, which is used for data dimensionality reduction to obtain a lower model computation load. Both the C4 and C5 layers consist of 128 channels with a convolution kernel size of 1 × 5. The number of channels is increased compared with the S3 layer. To satisfy the requirement of this operation, the feature map of each channel in the C4 layer is a combination of different feature maps extracted from S3. The S6 layer consists of 128 channels with a pooling kernel size of 1 × 2. The F7 layer is a flattening layer, which transforms the data into 1D data, thereby realizing the transition from the convolutional layer to the full connection layer. The F8 layer is a full connection layer, fully connected to the F7 layer, and, finally, the output layer outputs the SOM content prediction.

### 3.4. Model Evaluation

Using the mean square error (MSE) function as the loss function of the model, MSE represents the mean value of the squared difference between the predicted value and the true one. Within the range of [0, +∞), the larger the error, the bigger the value, and the formula is shown in Equation (2), where *t_ipre_* refers to the predicted value of the SOM content of Sample *i*, and *t_i_* stands for the true value of the SOM content of Sample *i*.

(2) $$\mathrm{MSE}=\frac{1}{n}\sum_{i=1}^{n}{\left(t_{ipre}-t_{i}\right)}^{2},$$

RMSE was used as the metric to evaluate the model performance. The calculation formula is shown in Equation (3), where *t_ipre_* represents the predicted value of the SOM content of Sample *i*, and *t_i_* refers to the true value of the SOM content for Sample *i*. The smaller the RMSE, the higher the accuracy of the model, which is more intuitive than MSE in terms of the order of magnitude.

(3) $$\mathrm{RMSE}=\sqrt{\frac{1}{n}\sum_{i=1}^{n}{\left(t_{ipre}-t_{i}\right)}^{2}},$$

The coefficient for the determination of R^2^ was introduced to assess the correlation between the predicted and true values. The calculation formula is shown in Equations (4) and (5), where *t_ipre_* denotes the predicted value of the SOM content of Sample *i*, and *t_i_* stands for the true value of the SOM content for Sample *i*. The higher the R^2^, the better the model fits.

(4) $$\bar{t}=\frac{1}{n}\sum_{i=1}^{n}t_{i}\;,$$

(5) $$R^{2}=1-\frac{\sum_{i=1}^{n}{\left(t_{i}-t_{ipre}\right)}^{2}}{\sum_{i=1}^{n}{\left(t_{i}-{\bar{t}}_{i}\right)}^{2}}\;,$$

The 563 sampling points were randomly divided into ten groups with each group containing 57 points; moreover, nine of them were used as the training set and one as the test set. The data from the training set and the test set were replaced after training once, and the mean value of the training results of the ten different datasets was taken for model evaluation based on a ten-fold, cross-validation method.

## 4. Results and Discussion

### 4.1. Results of Model Prediction

**Figure 8:**
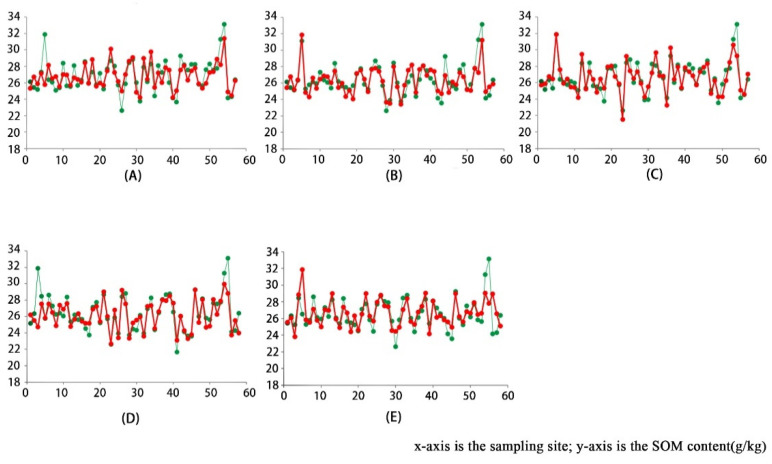
Model training results with different number of features (the red part refers to the model prediction, and the green part stands for the real value of SOM content): (**A**) inversion results based on 55-feature dataset; (**B**) inversion results based on 84-feature dataset; (**C**) inversion results based on 103-feature dataset; (**D**) inversion results based on 139-feature dataset; (**E**) inversion results based on 152-feature dataset.

**Figure 9:**
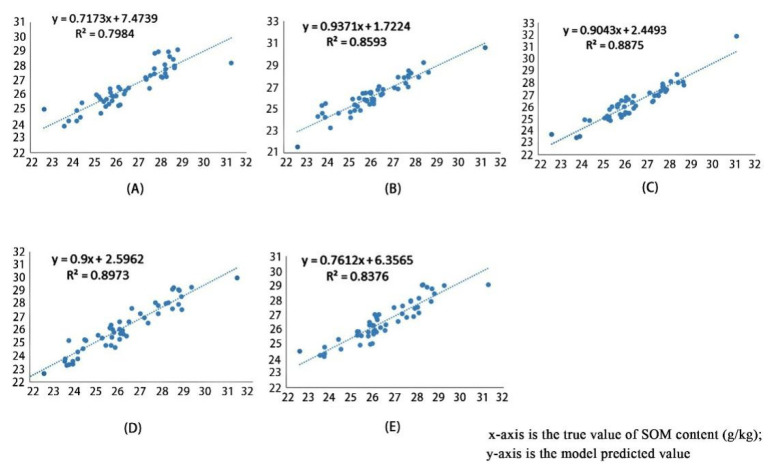
R^2^ of the true and predicted values with different number of features: (**A**) inversion results based on 55-feature dataset; (**B**) inversion results based on 84-feature dataset; (**C**) inversion results based on 103-feature dataset; (**D**) inversion results based on 139-feature dataset; (**E**) inversion results based on 152-feature dataset.

According to the results of feature selection, five sets of experiments were designed by setting up the dataset with different numbers of features. The results of the model training, as shown in Table 2, suggest that the inversion of the SOM content based on the deep learning model is feasible, and that the minimum RMSE can reach 3.354, while the R^2^ is 0.90, which proves the existence of a linear relationship between the predicted value and the real one. Moreover, the model has a prediction function. The different number of features are inverted within the same model with 1200 epochs, and the comparison of the model prediction effect is shown in Figure 8. The higher the overlap between the red line and the green line, the closer the model prediction to the true value. It can be seen that, with 152 features, the overall prediction result of the model is high, and the RMSE reaches 3.593, resulting in a certain gap between it and the real value. Similar prediction results are obtained based on 139 features, 103 features, and 84 features, respectively, and the RMSE values are close. The RMSE values, based on 55 features, are similar compared with the previous categories. However, the prediction values are concentrated around 26 g/kg, which is more single. Comparing the training results of R^2^ for each type of dataset (as shown in Figure 9), the lowest one is 55 features, which is consistent with the model training results shown in Figure 8. The only two types of R^2^ higher than 0.9 are 103 features and 139 features, which proves that the model’s prediction results of inverse performance under these two types of datasets are closely correlated with the real value of the SOM content. Taking a comprehensive consideration of the model training time shown in Table 2, with an increase in the number of features, the computational volume of the model increases, and the time consumed by the model training also increases. The SOM content inversion model established in this experiment is trained under 103 features, which can reduce the computation loading and shorten the model training time while still ensuring the accuracy and stability of the model.

**Figure 10:**
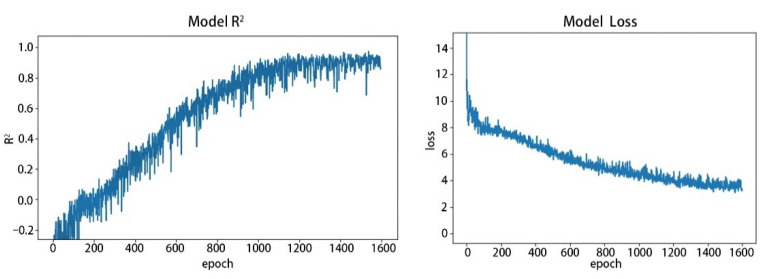
Model R^2^ curve and Model loss curve (The R^2^ and Model loss stabilizes at about 1200 epochs).

To describe the effect of the epochs on the model, the loss curve images of the model were plotted by using the 103-feature dataset. The results are shown in Figure 10. We also set up four sets of experiments according to Figure 10 for the 1000, 1200, 1400, and 1600 epochs, respectively, to evaluate the model effect comprehensively. The results are shown in Table 2.

### 4.2. Comparison with Other Models

Most of the current studies on the inversion of the SOM content of multispectral images rely on wavebands or their combinations to construct datasets and model them by linear regression analysis. In this experiment, based on the results of feature importance ranking, as shown in Figure 7, single or multiple features with top importance were selected as independent variables, and SOM content was selected as the dependent one for linear regression analysis. The data were also processed by regression analysis using the Support Vector Machines (SVM) algorithm, Random Forest (RF) algorithm, and the original 1D LeNet-5 model, the results of which are shown in Table 3. The linear regression analysis was implemented by using SPSS analysis software. The SVM algorithm was conducted based on the SVM. The SVR method was used in the Sklearn package and the Random Forest algorithm was implemented according to the Random Forest Regressor method in the sklearn package. Moreover, the 1D LeNet-5 model was built based on Keras in tensorflow. The training environment is the same as that of the improved model.

**Figure 11:**
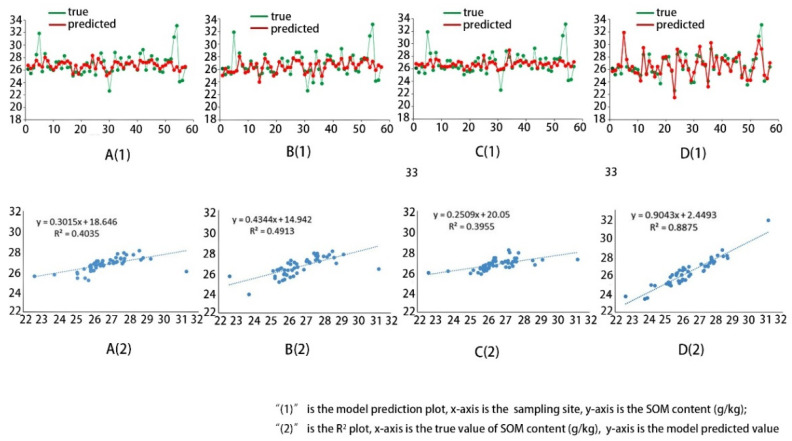
The results of different models: (**A**) support Vector Machines (SVM); (**B**) Random Forest (RF); (**C**) LeNet-5; (**D**) improved model.

As seen from the data in Table 3, the coefficient of the determination of the single-band inversion model is too low and insufficient to establish a correlation with SOM content. With an increase in independent variables, R^2^ shows an increasing tendency. However, too many independent variables are added instead of making the model’s R^2^ significantly lower. The R^2^ of the SVM algorithm is 0.40, and that of the RF algorithm is 0.49; moreover, both are higher than that of the linear regression method but lower than that of the improved model (R^2^ = 0.89). The training result R^2^ of the original 1D LeNet-5 model is improved compared with the linear regression analysis model; however, it is not as good as the other algorithms, as shown in Figure 11.

## 5. Discussion

SOM inversion by using multispectral data obtained from remotely sensed images is challenging. The spectral data errors may be caused by environmental effects (cloud cover, vegetation cover, etc.). Therefore, it is important to select images with bare soil periods and low cloudiness. In this experiment, we used Sentinel-2A images as the data source, which is the only optical data that contains three bands in the red-edge range (sensitive area for organic matter content). Moreover, we extracted the spectral data of the sampling sites as features and expanded the number of features by using mathematical transformation. The features were ranked in terms of importance based on the MLP algorithm. The result in Figure 7 shows that the features with higher importance were all double-band compositions, proving that the sensitivity of the spectral data to the SOM content could be effectively enhanced by using the mathematical transformation method. In addition, the SOM content is most sensitive in the near-infrared band (783~865 nm), followed by Band 6 (740 nm), Band 4 (660 nm), and Band 3 (560 nm), which is in agreement with the conclusions of other SOM content inversion experiments [12,13,14,25].

Based on the experimental results, it is found that the SOM inversion, by using the improved CNN model, is feasible. It can be seen in Table 2 that the 103-features (RMSE = 2.979, R^2^ = 0.8875) and 139-features (RMSE = 2.896, R^2^ = 0.8973) at 1200 epochs have the best results. Compared to the 103-features, the 139-feature dataset showed a small improvement in effectiveness, albeit an increase in runtime. However, 152-features are not as effective, with the inclusion of too many unimportant features affecting the judgment of the model. The highest RMSE was found for the result of the 55-feature dataset (RMSE = 3.362). When the number of features is too small, the number of features that can be extracted by the deep network when performing feature extraction is reduced, which causes errors in the prediction results. The lowest R^2^ is the result of a 103-feature dataset with 1000 epochs (R^2^ = 0.7686). The process of deep learning is the process by which the model learns, and the predicted value obtained is continuously approximated to the true value. When the number of epochs is insufficient, the model is not stabilized, resulting in a poor correlation between the predicted and true values. Figure 10 also shows that the model tends to stabilize at 1000 epochs, while the model is fully stable at about 1200 epochs.

Compared with statistical models, the advantage of deep learning models is that they have a stronger learning ability by training multiple layers of sample data. Due to the multi-layer neural network, the model can theoretically map many complex functions, which is highly adaptable. In this paper, the advantage of the improved model is mainly reflected by the improvement of R^2^. As can be seen from Figure 11, the fit of the improved model is significantly better than the other models. Compared with the linear regression model, SVM model, and RF model, the R^2^ of the improved model improved by 0.6908, 0.484, and 0.3962, respectively. The original one-dimensional LeNet-5 model does not show significant learning effects after several iterations because the depth is not able to meet the processing requirements of multi-feature datasets. After the model was analyzed, the number of convolutional layers was added to the model so that multiple feature extraction could be achieved, meaning that this deficiency was improved. In addition, by increasing the number of convolutional kernels, the features of the previous layer can be extracted more accurately and adequately to enhance the fitting ability of the model. After the improvement, the R^2^ of the model was improved by 0.492. It is noteworthy that the RMSE values of the models are low, as seen in Table 3. Through an analysis of the model and the data, we believe that this is caused by the high number of data, in which SOM lies within the average values of the sampling sites, and we plan to use more diverse soil samples in the next experiment for further study.

## 6. Conclusions

Changes in SOM content affect the spectral performance of soils. The CNN model implements feature extraction through a multilayer neural network to make the model predictions gradually approximate the true values, which provides a method for the inversion of soil organic matter from spectral reflectance. The soil organic matter content data obtained from the inversion can be used by resource environment/agriculture-related departments to assess soil nutrients, understand soil properties, and guide land classification management and crop planting planning.

This paper aims to construct an inverse model of SOM content based on multispectral image data through deep learning methods. Though deep learning models have been used previously to model hyperspectral image data, the number of multispectral image bands is small and therefore cannot meet the demand for the number of features required for the deep learning model. To solve this problem, we augmented the data by using a mathematical transformation method based on previous studies of multispectral images. In addition, we filtered out the features with higher importance by a feature selection method based on the MLP algorithm. Through the filtration of features, we not only lowered the noise generated by irrelevant features but also reduced the computational effort of the model. Moreover, through extensive experiments, we explored more suitable model parameters, including the number of convolution kernels and epochs, to further optimize the model.

It was found that the inversion of SOM content by amplified multispectral image data based on the deep learning method is feasible, and that the determination coefficients of the prediction results and the true value were significantly improved by the optimization of the model. Compared with hyperspectral images, multispectral images are easier to obtain with the reduced cost of data acquisition. With this method, the prediction of SOM content can be accomplished at a low cost. However, since the field sampling sites are all within Nong’an County, although multiple types of soil samples were collected, most of the SOM content was between 20 and 30 g/kg, resulting in the fact that the model’s improvement of RMSE values was not obvious. Because of this, in the subsequent experiments, it is expected that more diverse soil samples can be used to further test and improve the model.

## Figures and Tables

**Table 1:** Spectral properties of Sentinel-2 images.

Sentinel-2 Bands	Central Wavelength (μm)	Resolution (m)
Band 1—Coastal aerosol	0.443	60
Band 2—Blue	0.49	10
Band 3—Green	0.56	10
Band 4—Red	0.665	10
Band 5—Vegetation Red Edge	0.705	20
Band 6—Vegetation Red Edge	0.74	20
Band 7—Vegetation Red Edge	0.783	20
Band 8—NIR	0.842	10
Band 8A—Narrow NIR	0.865	20
Band 9—Water vapour	0.945	60
Band 10—SWIR-Cirrus	1.375	60
Band 11—SWR	1.61	20
Band 12—SWIR	2.19	20

**Table 2:** Comparison of the inversion effects of different parameters of soil organic matter content in the model.

Number of Features	Epochs	RMSE	R^2^	Model Training Time (min)	Single Sample Training Time (μs/Sample)
152	1200	2.909	0.8376	06:30.46	649
139	1200	2.896	0.8973	05:53.24	587
103	1600	3.333	0.8761	06:04.63	450
103	1400	3.113	0.8697	05:24.27	457
103	1200	2.979	0.8875	04:46.42	463
103	1000	3.202	0.7686	03:50.82	456
84	1200	3.248	0.8593	03:59.25	371
55	1200	3.362	0.7984	02:41.27	278

**Table 3:** Comparison of prediction effects of different models (X1 is B8 − B8a; X2 is the NDVI of B8a and B7; X3 is B5/B6; X4 is B4/B8a; X5 is the NDVI of B8a and B4, sorted by feature importance).

Model	RMSE	R^2^
X1	2.672	0.1245
X1 + X2 + X3	2.666	0.1967
SVM	2.471	0.4035
RF	2.577	0.4913
1D LeNet-5	2.730	0.3955
Improved Model	2.979	0.8875

## Data Availability

The data presented in this study are available on request from the corresponding author. The data are not publicly available due to private reasons.

## Supplementary Materials

The following supporting information can be downloaded at: https://www.mdpi.com/article/10.3390/s22207777/s1, Table S1: The original dataset with digital number.
